# Assessment of Neck Characteristics for Laryngeal Mask Airway Size Selection in Patients Who Underwent an Elective Ocular Surgery; A Cross-Sectional Study

**DOI:** 10.30476/BEAT.2022.94356.1338

**Published:** 2022-04

**Authors:** Omid Aghadavoudi, Hamidreza Shetabi, Hamid Saryazdi, Susan Babayi

**Affiliations:** 1 *Anesthesiology and Critical Care Research Center, Isfahan University of Medical Sciences, Isfahan, Iran*

**Keywords:** Laryngeal mask airway, Airway management, Anesthesia, Elective ocular surgery, Masks anesthesia

## Abstract

**Objective:**

To investigate the neck features for laryngeal mask airway (LMA) size selection.

**Methods:**

This cross-sectional study was conducted on 160 patients referred for elective surgeries to Feiz Hospital affiliated with Isfahan University of Medical Sciences, Isfahan, Iran (April 2016 to September 2018). Patients underwent ventilation using LMA whose size was determined through a weighted-based approach. All of the patients’ neck characteristics including circumference, thyromental distance, and opening mouth were measured. Ventilation factors were recorded including numbers of attempts for successful LMA insertion, quality of ventilation, and sealing.

**Results:**

Neck circumference and thyromental distance were significantly different with the size of LMA (*p*<0.0001 and *p*=0.005, respectively), but not mouth opening (*p*=0.21). Neck circumference, thyromental distance, and mouth opening were not significantly different with the times of insertion attempts (*p*>0.05 for all comparisons). However, the thyromental distance was significantly different with the quality ventilation status (*p*<0.0001). The total assessment of insertion attempts, ventilation efficacy and sealing was significantly different with the neck circumference (*p*<0.001), but thyromental distance did not show a significant difference (*p*>0.05).

**Conclusion:**

Findings demonstrated that neck circumference might be considered as an appropriate indicator for the selection of LMA size but neither the thyromental distance nor mouth opening. Further studies with a larger sample size are strongly recommended.

## Introduction

By the laryngeal mask airway (LMA) introduction in the 1980s, the practice of the anesthesia remarkably turned to the use of this device as a trustable and suitable alternative rather than the endotracheal tube in specific conditions regarding the type of the surgical procedure, the nature of the airway and patients’ individual characteristics [1]. Determinants such as the LMA size, the insertion technique, and cuff sealing would affect the proper and efficient use of this supraglottic device. However, the improper size of the LMA may lead to malpositioning and failure of ventilation [2, 3]. 

Although manufacturers represent guidelines for the practical use of these devices, selection of the appropriate size in order to ensure about the LMA performance and safety is still a great concern for anesthesiologists [4]. This approach is not always satisfactory because of the wide range of weights. Weighted-based LMA is the most common recommendation for LMA size determination due to the ease of the weight attributed identification to a specific size of LMA and the anatomical variations with more significance [5, 6]. To address this issue, efforts are in progress in order to determine the most practical strategy for the selection of LMA size [7, 8]. 

Varieties of factors such as thyromental distance, neck circumference, ideal body weight, and neck circumference to thyromental distance ratio have been estimated to be probably in association with the appropriate size of LMA, leading to the ultimate ventilation outcomes [3, 9-11]. This study aimed to assess the mean comparison of neck anatomical factors with the size of LMA and compare its outcomes with findings of weighted-based LMA selection.

## Materials and Methods

This cross-sectional study was conducted on 160 patients referred for ocular surgeries under general anesthesia to Feiz Hospital which is affiliated to Isfahan University of Medical Sciences, Isfahan, Iran from April 2016 to September 2018. Patients were consecutively selected and the study was conducted in accordance with the *Helsinki Declaration* statement. Moreover, this study was approved by the Ethics Committee of Isfahan University of Medical Sciences, Isfahan, Iran (No. IR.MUI.MED.REC.1397.246). The study protocol was explained to the participants and they were requested to sign the written consent form for participation in the study.

Patients with advanced surface ablation (ASA)-I or ASA-II conditions were included based on the American Society of Anesthesiologists physical status classification who were candidates for elective ocular operations requiring less than 2 hours of anesthesia. Furthermore, exclusion criteria were composed of unwillingness for participation in the study, inability to open the mouth for over 2 centimeters (cm), presentation of upper airway obstruction, being in the second or third trimester of pregnancy, supraglottic anatomical abnormalities, limitation to cervical extension due to any reason and type IV Mallampati classification.

Patients’ demographics including age, height, weight, body mass index (BMI), and ASA status were recorded in the study checklist. In addition, participants were physically examined to measure thyromental distance, the maximal capability of mouth opening, mallampati grading, and neck circumference.

The thyromental distance and width were measured between the thyroid gland and the mental epiphysis using a ruler with the minimal diagnosis of 0.5 mm. The LMA size was determined based on the patients’ body weight and LMA placement was performed by the anesthesiology resident. Afterward, patients were in a supine position, and hemodynamic variables including blood pressure, heart rate, and oxygen saturation were measured prior to the surgical procedure. Then, they were oxygenized and anesthesia was induced by agents’ injection includes 2 mg/kg of fentanyl, 0.02 mg/kg of midazolam, 2 mg/kg of propofol, and 0.5 mg/kg of atracurium. Finally, the LMA was placed and positive pressure ventilation was performed with the pressure of 15 cmH_2_O. The ventilator setting was adjusted at an inspiratory pressure of 10 cmH_2_O, inspiration duration of 1.6-2.5 seconds, respiratory rate of 8-12 per minute, inspiration to expiration ratio of 1:2, and 3 liters of fresh gas flow.

The ease of LMA placement was scored as 1) Easy: successful placement by once effort, 2) Relative: successful placement by twice efforts and 3) Difficult: more than twice efforts and/or neck repositioning requirement. Ventilation efficacy was determined based on the tidal volume as >8 cc/kg which was defined as an excellent and 4-8 cc/kg as an acceptable and <4 cc/kg as low. Laryngeal mask sealing was the rater evaluation justified by the noise heard through the LMA or by the auscultation of the neck using a stethoscope. Any failure of LMA placement leading to intubation and complications includes laryngospasm, hypoxia, and aspiration were recorded as well. Within 5 minutes, all of the patients were interviewed by assessing complications including dysphagia.

*Statistical Analysis*

All continuous and categorical variables were expressed as mean±standard deviation (M±SD) and numbers (percentages), respectively. The Kolmogorov–Smirnov test was used to determine the normality of data. The comparison of continuous and categorical variables between groups (more than two groups) was analyzed using one-way analysis of variance (ANOVA)/Kruskal-Wallis following post-hoc Tukey/Mann-Whitney U tests, and Chi-square tests, respectively. Moreover, the differences of continuous variables between the two groups were calculated using the Independent sample t-test. The statistical analyses were conducted using SPSS software version 20.0 for Windows (IBM/SPSS Inc., New York, USA). Statistical significance was assumed at *p*-values less than 0.05.

## Results

The patients’ mean age was 61.7±13.0 years and 71 (44.4%) were female. The mean neck circumference and mouth opening were 38.6±4.6 cm and 5.1±0.9 cm, respectively. Based on patients’ weight, the size of LMA in 131 (63.1%) of the patients was four, in 39 (24.4%) was five, and in 20 (12.5%) was three. Ventilation efficacy in most of the patients was excellent (54.2%) or acceptable (43.9%). Also, in 131 (83.1%) of studied patients, the first insertion attempt was successful (Table 1).

**Table 1:** The demographic and clinical characteristics of studied patients

**Number**	**160**
Age (year)	61.7±13.0
Sex	
Male	89 (55.6)
Female	71 (44.4)
Height (cm)	165.9±10.4
Weight (kg)	67.3±14.0
BMI^a^ (kg/m2)	24.1±3.4
ASA^b^ PS	
I	78 (48.8)
II	82 (51.2)
Mallampati class	
1	16 (10)
2	121 (75.6)
3	23 (14.4)
Thyromental distance (cm)	6.6±1.2
Neck circumference (cm)	38.6±4.6
Mouth opening (cm)	5.1±0.9
Weighted-based LMA^c^	
3 (30-50 kg)	20 (12.5)
4 (50-70 kg)	101 (63.1)
5 (>70 kg)	39 (24.4)
Efficacy of ventilation	
Tidal volume ≥8 mL/kg (excellent)	85 (54.2)
4≥ Tidal volume <8 mL/kg (acceptable)	69 (43.9)
Tidal volume <4 (low)	3 (1.9)
Number of insertion attempts	
One time	133 (83.1)
1-2 times	23 (14.4)
>2 times	4 (2.5)
Sealing	141 (88.1)
Data are presented as mean±SD^d^, or number (%).	

^a^BMI: Body Mass Index; ^b^ASA: Advanced Surface Ablation; ^c^LMA: Laryngeal Mask Airway; ^d^SD: Standard Deviation.

Table 2 represents the mean comparison of neck anatomical features with the weighted based LMA size selection. As shown, two-by-two comparisons showed that neck circumference in patients who received size 5 of LMA was significantly more than patients who received sizes 3 (*p*≤0.001) or 4 (*p*=0.003). Also, the mean of thyromental distance in patients with LMA 5 was 7.1 which was significantly more than the patients who received with sizes 3 (*p*=0.001) or 4 (*p*=0.002). Similarly, the mean of weight in patients with LMA 5 was 83.9±9.6 kg which was significantly higher than those received with sizes 3 (47.6±4.2 kg) (*p*<0.001) or 4 (64.6±9.1 kg) (*p*=0.004). However, the mean of mouth opening in studied patients was not significantly in regard to LMA size (*p*=0.212).

**Table 2:** Comparison of neck demographic factors in studied patients by weighted based LMA

	**Weighted based LMA** ^a^ ** groups**			***p*** value*
	**3 (30-50 kg), n=20** **Mean (SD)**	**4 (50-70 kg), n=101** **Mean (SD)**	**5 (>70 kg), n=39** **Mean (SD)**	
Weight (kg)	47.6 (4.2)	64.6 (9.1)	83.9 (9.6^b)^	<0.001
Neck circumference, cm	33.2 (7.6)	38.1 (2.8)	42.4 (3.1^b)^	<0.001
Thyromental distance	6.3 (1.2)	6.4 (1.0)	7.1 (1.4^b)^	0.005
Mouth opening, cm	5.0 (1.0)	5.1 (0.9)	5.4 (0.9)	0.212

*p values calculated using one way of ANOVA and post-hoc Tukey test; ^a^LMA: Laryngeal Mask Airway; ^b^Significant difference with first and second groups.

Table 3 shows the mean differences between neck demographic factors and success insertion attempts among the studied patients. Among studied neck demographic factors only patients weight was significantly different with the first time successful insertion as compared to more than one attempts for successful insertion (*p*=0.005) but neck circumference, thyromental distance and mouth opening were not significantly different with the times of insertion attempts (*p*>0.05). 

**Table 3:** Comparison of neck demographic factors in studied patients by Success insertion attempts

	**Successful insertion attempts**		***p*** ** value** ^a^
	**First attempt success, n=20** **Mean (SD)**	**More than one attempts, n=101** **Mean (SD)**	
Weight (kg)	65.9 (13.1)	74.2 (16.4)	0.005
Neck circumference, cm	38.3 (4.7)	39.9 (4.0)	0.088
Thyromental distance	6.6 (1.1)	6.7 (1.6)	0.811
Mouth opening, cm	5.1 (0.9)	5.3 (1.1)	0.247

^a^ *p* values calculated using Independent sample t test

Table 4 shows the assessments of neck demographic factors and efficacy of ventilation among studied patients. Patients’ weight, body mass index (BMI), neck circumference and mouth opening were not significantly different with ventilation efficacy status (*p*>0.05), while two-by-two comparisons showed that thyromental distance in patients with acceptable ventilation status was significantly more than those patients with excellent or low quality ventilation status (*p*<0.001 for both comparisons).

**Table 4:** Comparison of neck demographic factors in studied patients by efficacy of ventilation

	**Efficacy of ventilation**			***p*** ** value**
	**Excellent, n=85** **Mean (SD)**	**Acceptable, n=68** **Mean (SD)**	**Low, n=3** **Mean (SD)**	
Weight (kg)	65.9 (13.3)	69.5 (13.8)	62.0 (10.5)	0.259^a^
Body mass index (BMI) (kg/m^2^)	24.1 (1.3)	24.2 (1.4)	22.9 (1.2)	0.525^b^
Neck circumference, cm	38.3 (4.0)	38.9 (5.4)	37.0 (5.0)	0.154^a^
Thyromental distance	6.2 (1.1)	7.1 (1.1^c)^	6.2 (0.8)	<0.001^a^
Mouth opening, cm	5.1 (1.0)	5.1 (0.9)	4.6 (0.6)	0.673^a^

^a^ *p* values calculated using Kruskal–Wallis test; ^b^*p* value calculated using ANOVA test; ^c^Significant difference with “Excellent” and “Low” groups.

Table 5 represents the comparison of neck circumference and thyromental distance mean with LMA based on insertion attempts, ventilation efficacy and sealing. All of the assessments revealed significant mean difference with the neck size (*p*<0.001), while thyromental assessments showed no significant statistical difference (*p*>0.05). 

**Table 5:** Mean comparison between neck circumference and thyromental distance with LMA based on insertion attempts, ventilation efficacy, and sealing

**Weighted based LMA**	**n**	**Neck circumference, cm**		***p*** ** value**	**Thyromental distance**		***p*** ** value** ^ a^
		**mean (SD)**	**95%CI**		**mean (SD)**	**95%CI**	
First/Excellent/Sealing							
3 (30-50 kg)	10	33.6 (1.8)	32.3-34.9		5.9 (1.1)	5.1-6.7	
4 (50-70 kg)	46	37.9 (3.3)	36.9-38.9	<0.001	6.1 (0.9)	5.8-6.4	0.748
5 (>70 kg)	8	43.9 (2.9)	41.5-46.4		6.4 (1.4)	5.2-7.5	
First/Excellent or Acceptable/Sealing							
3 (30-50 kg)	15	32.2 (8.3)	27.6-36.8		6.1 (1.2)	5.5-6.9	
4 (50-70 kg)	81	38.1 (2.8)	37.5-38.8	<0.001	6.4 (1.0)	6.2-6.7	0.105
5 (> 70 kg)	21	42.6 (3.2)	41.2-44.1		6.9 (1.2)	6.4-7.5	
First or two/Excellent or Acceptable/Sealing							
3 (30-50 kg)	16	32.1 (8.0)	27.8-36.3		6.1 (1.1)	5.5-6.7	
4 (50-70 kg)	91	38.2 (2.8)	37.6-38.7	<0.001	6.4 (1.0)	6.2-6.6	0.052
5 (>70 kg)	30	42.7 (2.9)	41.6-43.8		7.0 (1.4)	6.5-7.6	

^a^ *p* values calculated using Kruskal–Wallis test.

In studied patients, only four complications were occurred includes complications in patients with LMA size 4 (two cough and one sore throat), and one cough in patients with LMA size 5.

## Discussion

Results obtained from the present study demonstrated that neck circumference might be an appropriate indicator for the selection of LMA size in patients who underwent elective ocular surgeries, however, the thyromental distance and mouth opening did not show a significant difference with LMA size.

The laryngeal mask airway is the most popular supraglottic airway used for general anesthesia induction due to elective surgeries that can appropriately keep spontaneous breathing during the surgical procedure. Beyond this advantage, the improper placement of LMA due to any reason can cause notable complications [12]. Previous studies have presented limited values of one screening test for the selection of appropriate LMA size to achieve the ultimate quality of ventilation [13]. Thus a combination of risk assessments and individual tests seems to be more efficient in comparison with each of the tests alone. Therefore scoring systems such as the El-Ganzouri or Wilson scores were introduced [14, 15]. Although these tests evaluate multiple risk factors, they are considerably time-consuming. On the other hand, the easiest means for the determination of appropriate LMA size, patient’s body weight was not necessarily accompanied by successful outcomes. In the current study, we selected neck-related features as the factors easily available but not time-consuming to measure the assessment of their value for LMA size selection.

We used three factors of attempts’ number for successful LMA insertion, the quality of ventilation, and presence of appropriate sealing as the indicators presenting efficacy of the placed LMA for the patients. Observing the mentioned factors in the selected size of LMA through the weighted based LMA approach and assessing its mean difference with neck-related features represented that the neck circumference is totally related to the successful outcomes of LMA placement includes the attempts’ number for successful placement, the quality of ventilation and sealing. This is while mere assessment of the attempts to achieve successful insertion and quality of ventilation were not statistically different with the neck circumference. 

Although neck circumference alone may not necessarily present the amount of the soft tissue surrounding all regions of the neck [9], Horner *et al*., assessed the soft tissue surrounding collapsible segments of the neck tissue through magnetic resonance imaging (MRI) and presented considerable higher amounts of fat pad among obese patients as compared to the general population [16]. This finding has been presented by other studies as well, because most of the challenges for intubation are attributed to obese cases that have short and thick necks [17]. Kim *et al*., conducted a study regarding the LMA size selection based on factors including neck circumference, BMI, and thyromental distance and eventually their ratio. Contrary to our study, they showed that neck circumference alone cannot appropriately determine the size of LMA [9]. This statement was confirmed by Seet *et al*., as well assessing both Supreme and Proseal laryngeal masks [18]. It is while other studies by Katsiampoura *et al*., [17] and Ahn *et al*., [19] represented the association of the neck circumference with the LMA size and also ventilation quality. 

Thyromental distance evaluations revealed no significant difference with the assessed indicators in general, while the efficacy of ventilation was merely different with the thyromental distance as patients with excellent ventilation had lower thyromental distance. Contrary to our findings, Weng *et al*., presented that thyromental distance was significantly associated with the number of attempts required for successful insertion of LMA [3]. Their findings were compatible with other studies regardless of the LMA type, whether Supreme or Proseal [18, 20, 21]. The other assessment regarded ventilation quality that was statistically different with the thyromental distance and confirmed by other authors’ presentations, though they presented diverse factors as the indicator of ventilation quality [18, 22].

The rater assessed factor was mouth opening difference with ventilation efficacy, successful insertion attempts, and weighted-based selected LMA. All of the entities revealed no remarkable differences. Although we have not found any significant difference, based on our research in the literature, mouth opening has not been considered as a factor for the selection of LMA size.

We found that neck circumferences might be an appropriate indicator for the selection of LMA size but neither the thyromental distance nor mouth opening. Further clinical trial studies are strongly recommended.

## Declarations

### Ethics approval and consent to participate:

This study conforms to the *Declaration of Helsinki* regarding research involving human subjects and is approved by Ethics Committee of Isfahan University of Medical Sciences, Isfahan, Iran (IR.MUI.MED.REC.1397.246). All participants signed the written informed consents form.

### Consent for publication:

Not applicable.

### Conflict of Interest:

The authors declare that they have no conflict of interest.

### Funding:

None.

### Author contributions:

All named authors meet the International Committee of Medical Journal Editors (ICMJE) criteria for authorship for this article, take responsibility for the integrity of the work as a whole, and have given their approval for this version to be published.

### Acknowledgments

Authors would like to express their deep gratitude towards participants who provided us with their precious assistance in performing this study.
